# Supplementary material for: A Comprehensive and Versatile Multimodal Deep‐Learning Approach for Predicting Diverse Properties of Advanced Materials
Source: Adv Sci (Weinh). 2023 Jun 26;10(24):2302508. doi: 10.1002/advs.202302508 (PMC10460884; doi:10.1002/advs.202302508)
Supplement: Supplementary file 1 — Supporting Information [file ADVS-10-2302508-s001.pdf]

ADVANCED SCIENCE
Open Access

# Supporting Information

for *Adv. Sci.*, DOI 10.1002/advs.202302508

A Comprehensive and Versatile Multimodal Deep-Learning Approach for Predicting Diverse Properties of Advanced Materials

*Shun Muroga, Yasuaki Miki and Kenji Hata**

Supporting Information

**A Comprehensive and Versatile Multimodal Deep Learning Approach for Predicting Diverse Properties of Advanced Materials**

*Shun Muroga, Yasuaki Miki, and Kenji Hata**

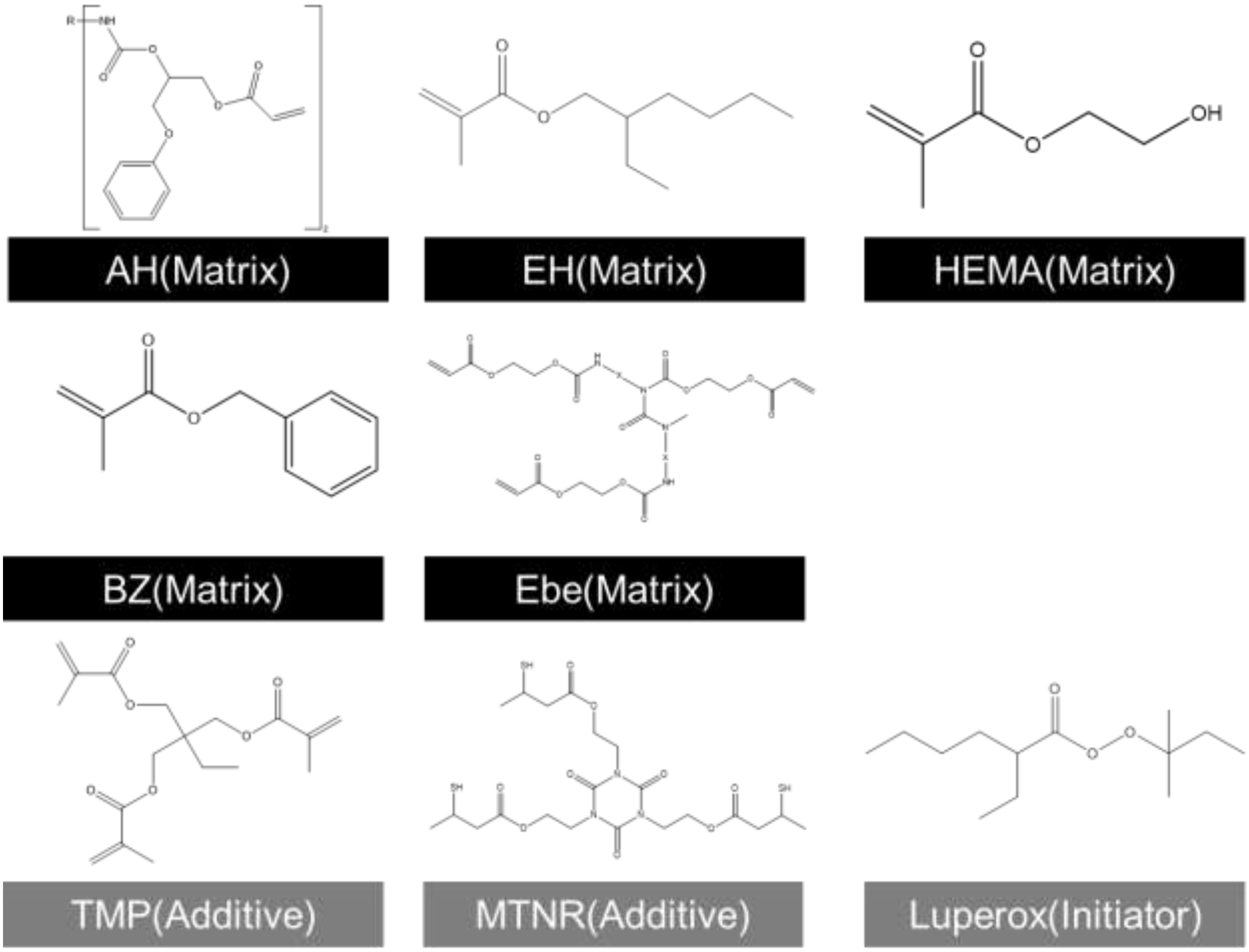


**Figure S1.** Schematics of molecular structures of matrix monomers and additives used in this study.

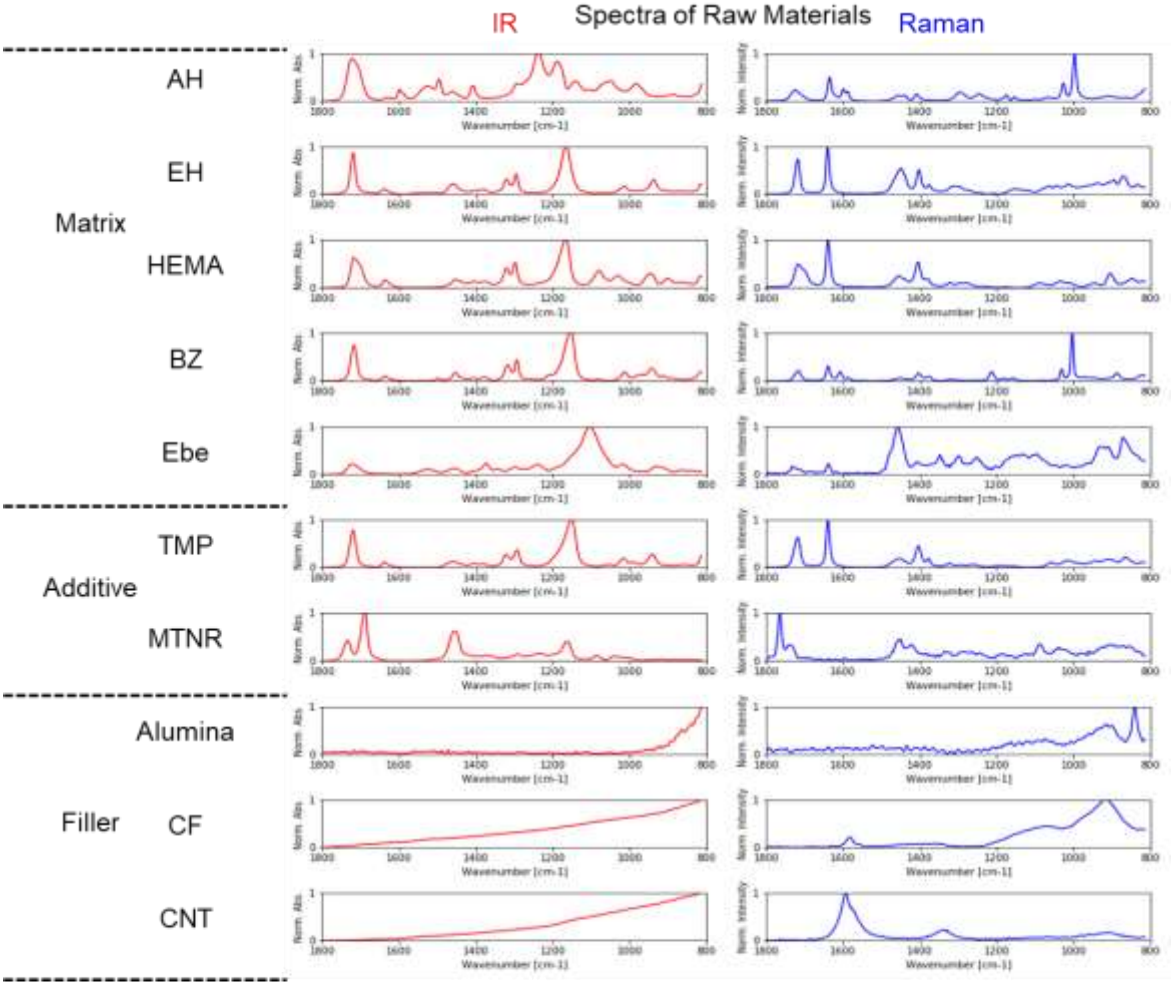


**Figure S2.** IR and Raman spectra of individual raw matrix monomers, additives, and fillers without any dispersion and curing.

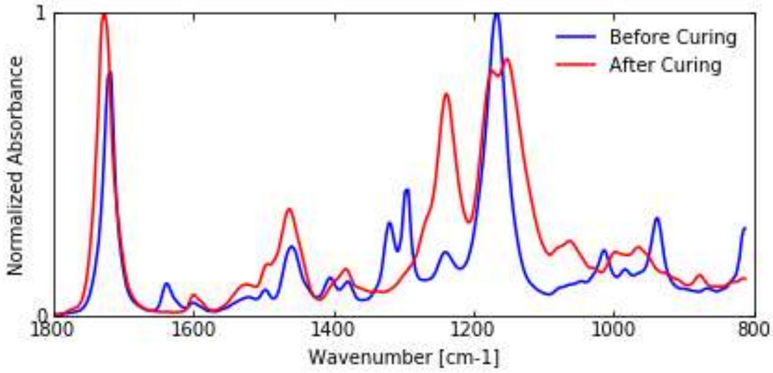


**Figure S3.** IR spectra of the polymer composite before and after curing. The composition of the polymer composite is AH20/EH80/CF20/Alumina20/CNT0.1.

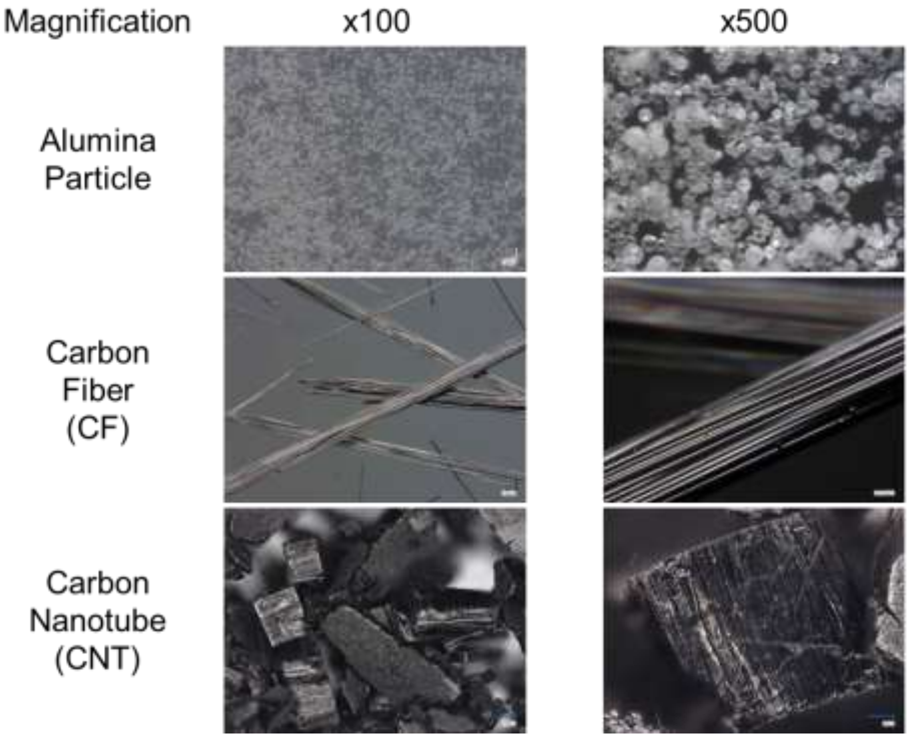

**Figure S4.** Optical microscope images of raw fillers without any dispersion and curing.

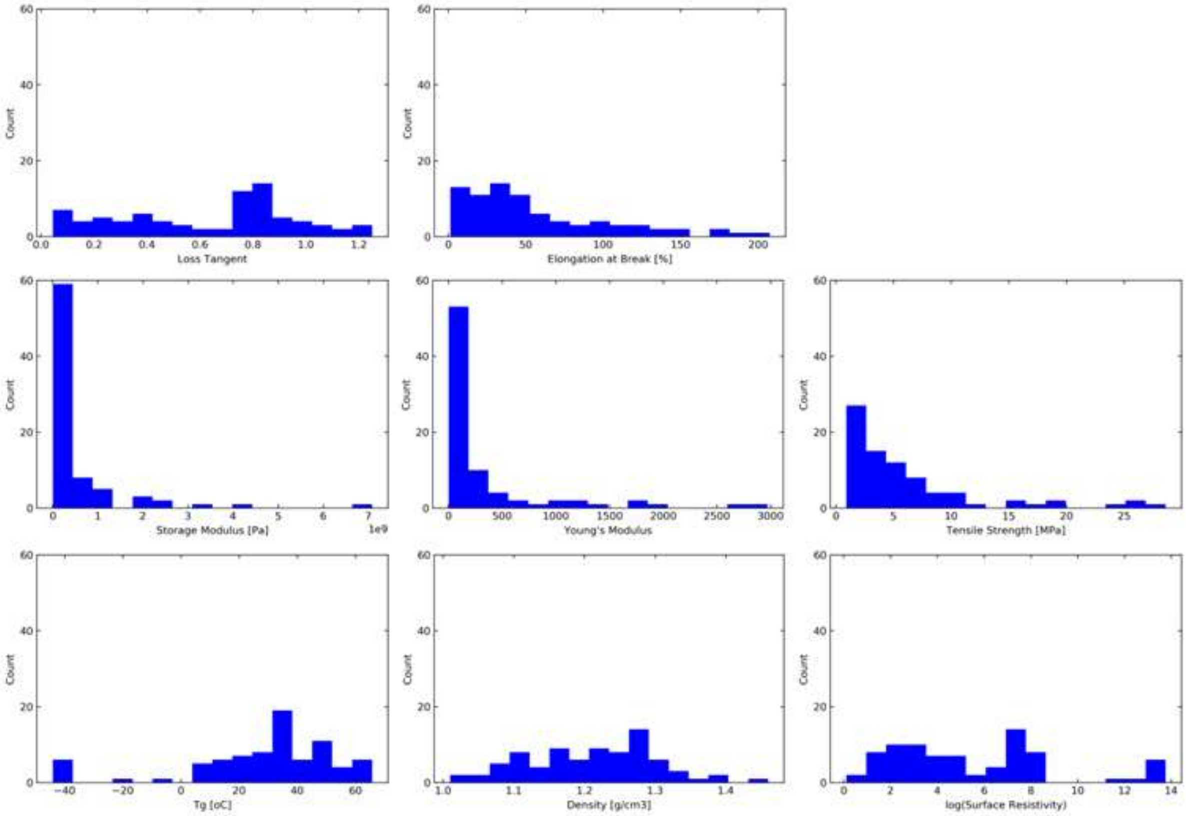


**Figure S5.** Distributions of the eight physical properties of the 80 polymer composites.

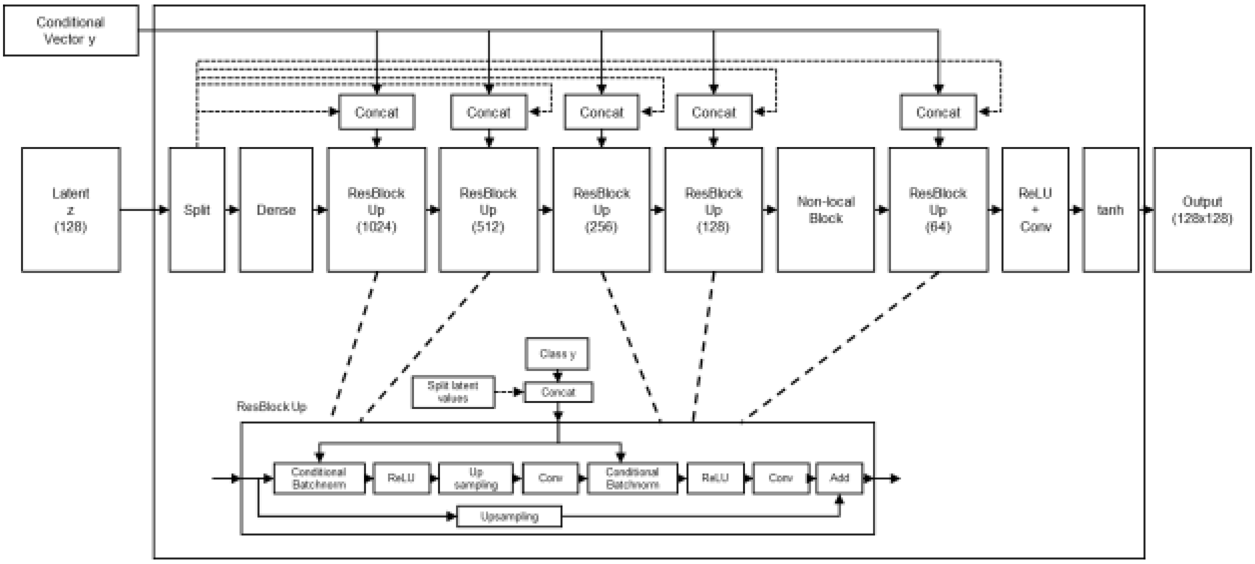

**Figure S6.** Architecture of BigGAN generator (128x128) for OM-GAN in this study.

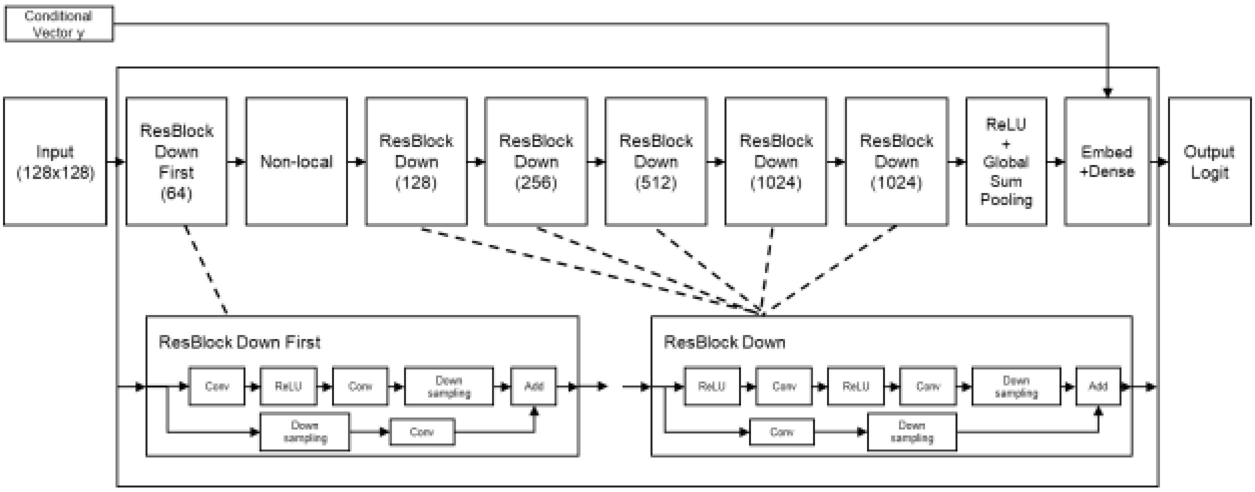


**Figure S7.** Architecture of BigGAN discriminator (128x128) for OM-GAN in this study.

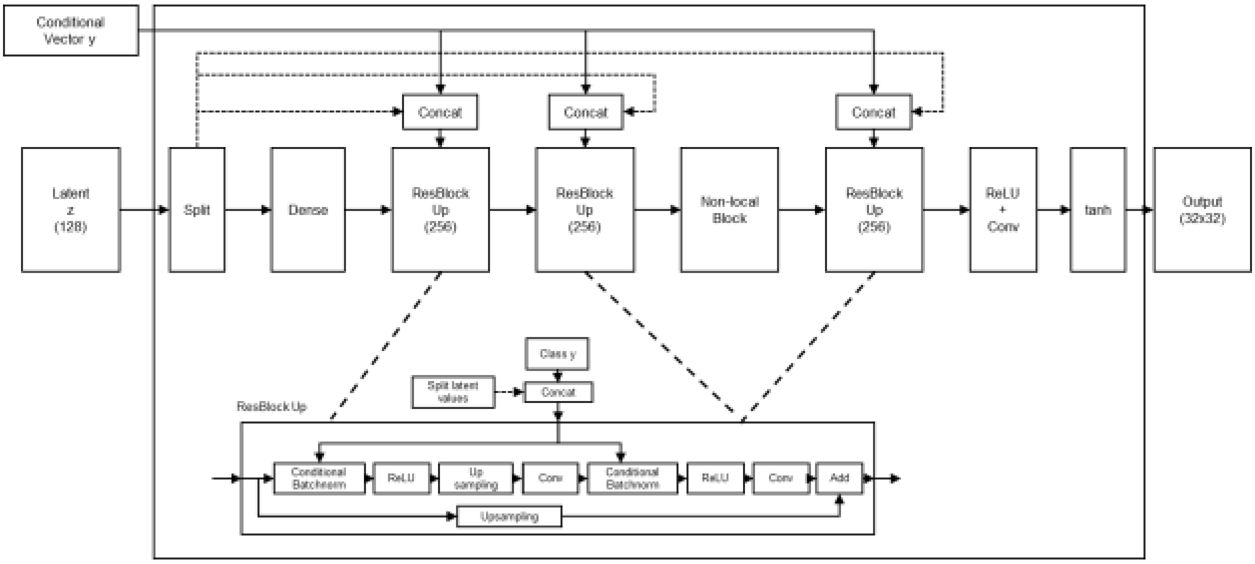


**Figure S8.** Architecture of BigGAN generator (32x32) for IR-GAN and Raman-GAN in this study.

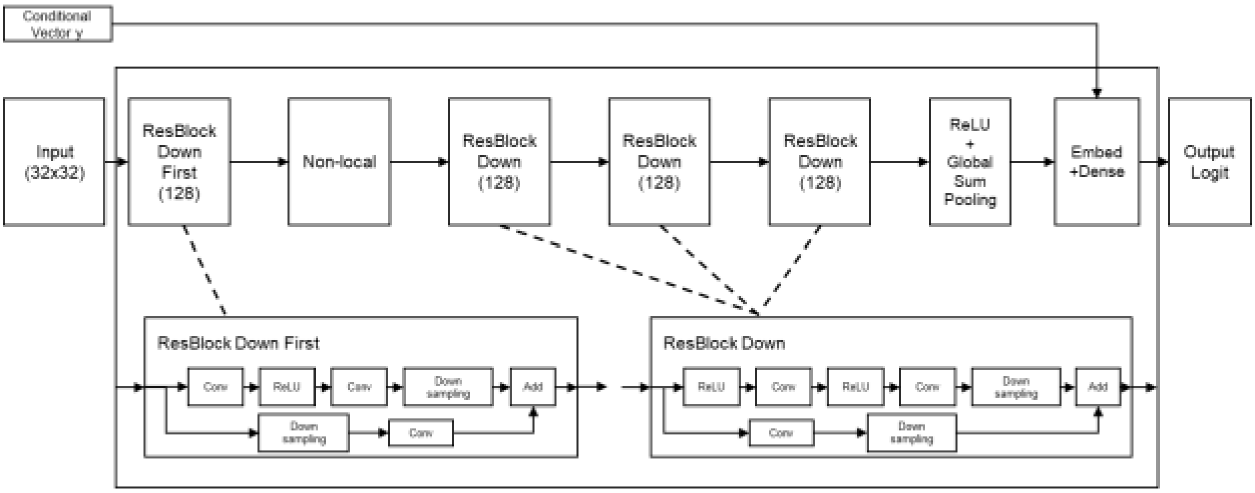


**Figure S9.** Architecture of BigGAN discriminator (32x32) for IR-GAN and Raman-GAN in this study.

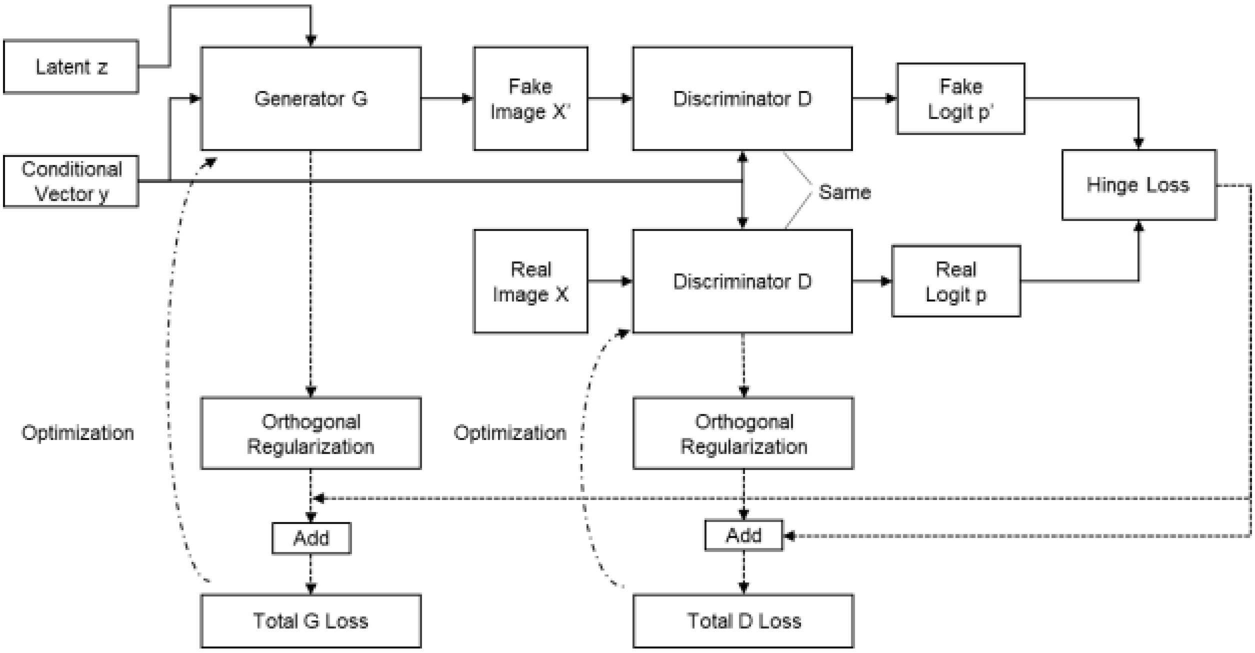

**Figure S10.** Training protocol of BigGAN generator and discriminator.

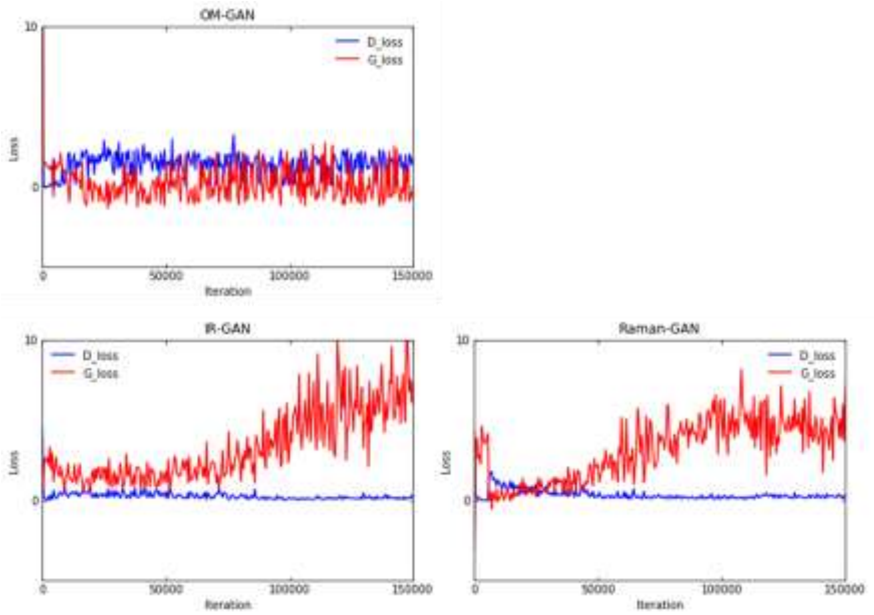


**Figure S11.** Loss histories of OM-GAN, IR-GAN, and Raman-GAN.

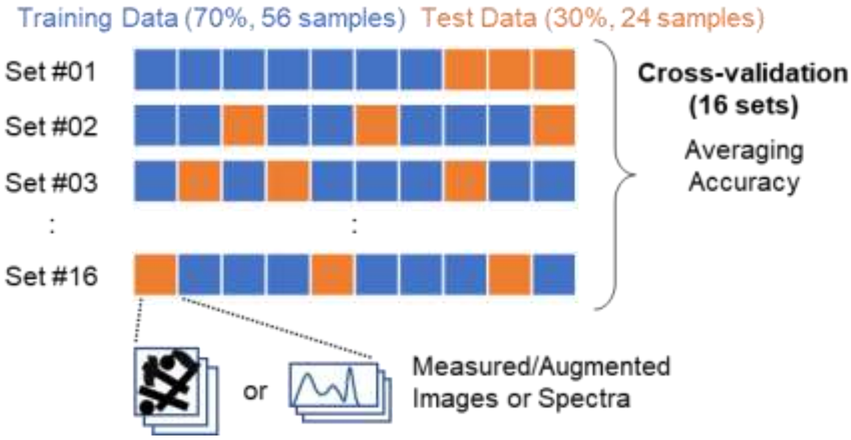


**Figure S12.** Schematic of cross-validation for optimizing multimodal deep learning model in this study.

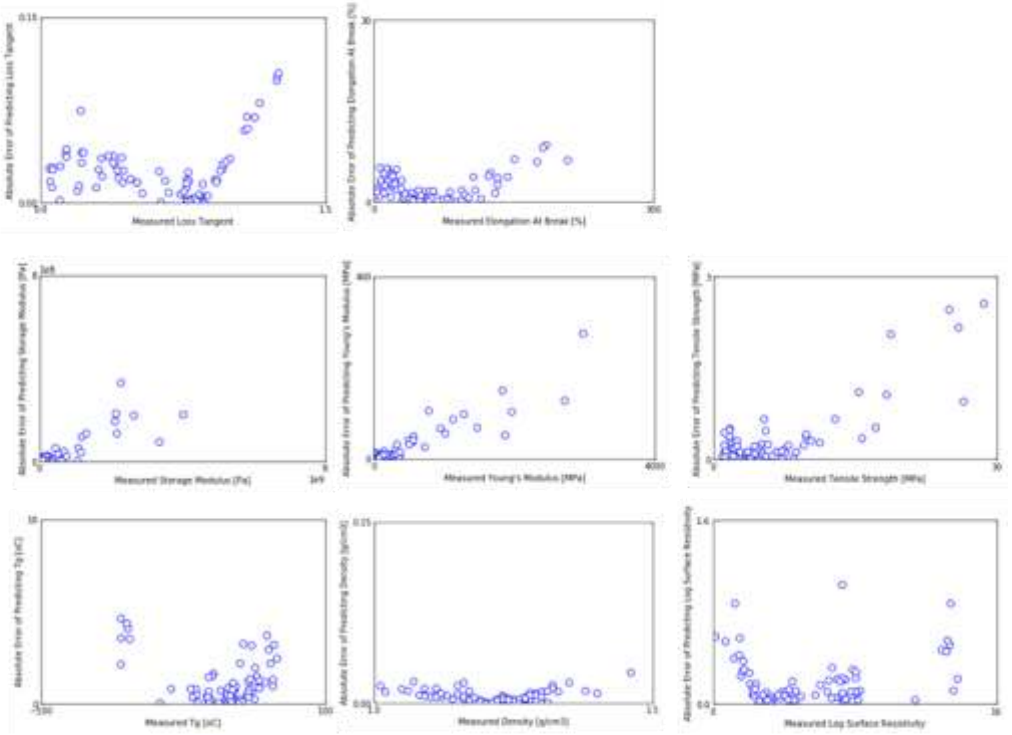

**Figure S13.** Absolute errors of the predictions of the eight physical properties.

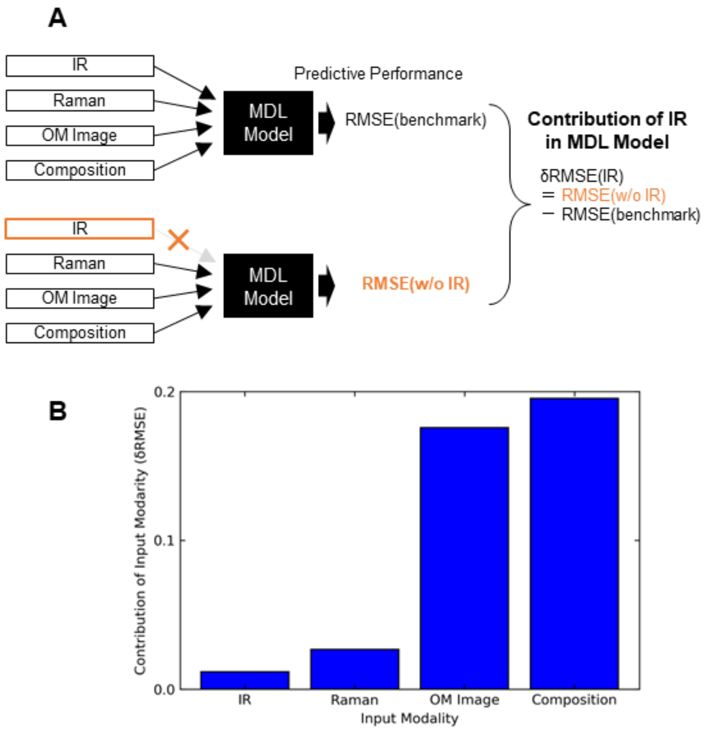


**Figure S14.** (A) Schematic of calculating contribution of each input modality. (B)Contribution plot of input modality in multimodal deep learning. δRMSE is the difference of RMSE between the two multimodal deep learning models with and without removing specific input modality. The detailed statistics are shown in Table S3.

**Table S1.** Compositional information of 80 polymer composite samples used in this study.

| AH | EH | HEMA | BZ | Ebe | TMP | MTNR | CNT | Alumina | CF |
|---|---|---|---|---|---|---|---|---|---|
| 0 | 80 | 0 | 0 | 20 | 0 | 0 | 0.2 | 0 | 10 |
| 0 | 80 | 0 | 0 | 20 | 0 | 0 | 0.2 | 0 | 0 |
| 0 | 80 | 0 | 0 | 20 | 0 | 0 | 0.2 | 0 | 20 |
| 0 | 60 | 0 | 0 | 40 | 0 | 0 | 0.1 | 20 | 0 |
| 0 | 41 | 0 | 31 | 28 | 0 | 0 | 0.1 | 10 | 0 |
| 0 | 42 | 0 | 30 | 28 | 0 | 0 | 0.1 | 10 | 10 |
| 0 | 40 | 0 | 0 | 60 | 0 | 0 | 0.5 | 0 | 0 |
| 0 | 80 | 0 | 0 | 20 | 0 | 0 | 0.1 | 10 | 10 |
| 0 | 80 | 0 | 0 | 20 | 0 | 0 | 0.2 | 10 | 10 |
| 0 | 42 | 0 | 30 | 28 | 0 | 0 | 0.1 | 0 | 10 |
| 0 | 60 | 0 | 0 | 40 | 0 | 0 | 0.1 | 10 | 10 |
| 0 | 60 | 0 | 0 | 40 | 0 | 0 | 0.5 | 10 | 10 |
| 0 | 42 | 0 | 31 | 28 | 0 | 0 | 0.2 | 10 | 10 |
| 0 | 60 | 0 | 0 | 40 | 0 | 0 | 0.1 | 20 | 20 |
| 0 | 80 | 0 | 0 | 20 | 0 | 0 | 0.2 | 20 | 20 |
| 0 | 60 | 0 | 0 | 40 | 0 | 0 | 0.5 | 20 | 20 |
| 0 | 56 | 0 | 30 | 14 | 0 | 0 | 0.1 | 10 | 10 |
| 0 | 42 | 0 | 30 | 28 | 0 | 0 | 0.5 | 20 | 20 |
| 0 | 0 | 0 | 0 | 100 | 0 | 0 | 0.1 | 10 | 10 |
| 0 | 80 | 0 | 0 | 20 | 0 | 0 | 0.5 | 20 | 20 |
| 0 | 56 | 0 | 30 | 14 | 0 | 0 | 0.5 | 10 | 10 |
| 0 | 0 | 0 | 0 | 100 | 0 | 0 | 0 | 0 | 20 |
| 0 | 40 | 0 | 0 | 60 | 0 | 0 | 0 | 0 | 20 |
| 0 | 56 | 0 | 30 | 14 | 0 | 0 | 0.2 | 20 | 20 |
| 0 | 42 | 15 | 15 | 28 | 0 | 0 | 0.5 | 20 | 20 |
| 0 | 0 | 0 | 0 | 100 | 0 | 0 | 0.5 | 20 | 20 |
| 0 | 20 | 0 | 0 | 80 | 0 | 0 | 0.5 | 20 | 20 |
| 0 | 52 | 0 | 30 | 17 | 0 | 1 | 0.5 | 20 | 20 |
| 0 | 56 | 0 | 30 | 14 | 0 | 0 | 0.5 | 20 | 20 |
| 20 | 0 | 0 | 0 | 80 | 0 | 0 | 0.1 | 20 | 20 |
| 20 | 0 | 0 | 0 | 80 | 0 | 0 | 0.5 | 20 | 20 |
| 0 | 52 | 0 | 30 | 17 | 1 | 0 | 0.5 | 20 | 20 |
| 0 | 52 | 15 | 15 | 17 | 0 | 1 | 0.5 | 20 | 20 |
| 20 | 80 | 0 | 0 | 0 | 0 | 0 | 0.3 | 20 | 0 |
| 20 | 80 | 0 | 0 | 0 | 0 | 0 | 0.1 | 5 | 5 |
| 20 | 80 | 0 | 0 | 0 | 0 | 0 | 0.1 | 10 | 5 |
| 20 | 80 | 0 | 0 | 0 | 0 | 0 | 0.1 | 10 | 10 |

| | | | | | | | | | |
|---|---|---|---|---|---|---|---|---|---|
| 0 | 56 | 30 | 0 | 14 | 0 | 0 | 0.2 | 10 | 10 |
| 20 | 80 | 0 | 0 | 0 | 0 | 0 | 0.1 | 15 | 10 |
| 0 | 52 | 30 | 0 | 17 | 0 | 1 | 0.5 | 20 | 20 |
| 20 | 80 | 0 | 0 | 0 | 0 | 0 | 0.1 | 5 | 10 |
| 20 | 80 | 0 | 0 | 0 | 0 | 0 | 0.1 | 20 | 10 |
| 20 | 80 | 0 | 0 | 0 | 0 | 0 | 0.3 | 20 | 20 |
| 20 | 80 | 0 | 0 | 0 | 0 | 0 | 0.5 | 20 | 10 |
| 20 | 80 | 0 | 0 | 0 | 0 | 0 | 0.1 | 10 | 15 |
| 0 | 52 | 15 | 15 | 17 | 1 | 0 | 0.5 | 20 | 20 |
| 20 | 80 | 0 | 0 | 0 | 0 | 0 | 0.5 | 10 | 10 |
| 20 | 80 | 0 | 0 | 0 | 0 | 0 | 0.1 | 10 | 20 |
| 20 | 80 | 0 | 0 | 0 | 0 | 1 | 0.5 | 20 | 20 |
| 40 | 0 | 0 | 0 | 60 | 0 | 0 | 0.1 | 10 | 10 |
| 20 | 80 | 0 | 0 | 0 | 0 | 0 | 0.5 | 20 | 20 |
| 20 | 80 | 0 | 0 | 0 | 0 | 0 | 0.5 | 10 | 20 |
| 21 | 79 | 0 | 0 | 0 | 0 | 1 | 0.5 | 0 | 20 |
| 40 | 0 | 0 | 0 | 60 | 0 | 0 | 0.1 | 20 | 20 |
| 18 | 53 | 0 | 30 | 0 | 0 | 0 | 0.1 | 20 | 0 |
| 18 | 53 | 0 | 30 | 0 | 0 | 0 | 0.3 | 20 | 0 |
| 0 | 52 | 30 | 0 | 17 | 1 | 0 | 0.5 | 20 | 20 |
| 14 | 56 | 15 | 15 | 0 | 0 | 1 | 0.1 | 20 | 0 |
| 20 | 80 | 0 | 0 | 0 | 1 | 0 | 0.5 | 20 | 20 |
| 20 | 80 | 0 | 0 | 0 | 0 | 0 | 0.5 | 0 | 20 |
| 20 | 80 | 0 | 0 | 0 | 1 | 0 | 0.5 | 0 | 20 |
| 0 | 52 | 0 | 30 | 17 | 5 | 0 | 0.5 | 20 | 20 |
| 20 | 80 | 0 | 0 | 0 | 0 | 0 | 0.5 | 30 | 30 |
| 14 | 56 | 15 | 15 | 0 | 0 | 1 | 0.1 | 0 | 20 |
| 14 | 56 | 15 | 15 | 0 | 0 | 1 | 0.3 | 0 | 20 |
| 14 | 56 | 15 | 15 | 0 | 1 | 0 | 0.1 | 20 | 0 |
| 0 | 52 | 15 | 15 | 17 | 5 | 0 | 0.5 | 20 | 20 |
| 14 | 56 | 15 | 15 | 0 | 1 | 0 | 0.3 | 20 | 0 |
| 18 | 52 | 30 | 0 | 0 | 0 | 0 | 0.1 | 10 | 10 |
| 0 | 53 | 30 | 0 | 17 | 5 | 0 | 0.5 | 20 | 20 |
| 20 | 80 | 0 | 0 | 0 | 5 | 0 | 0.5 | 20 | 20 |
| 20 | 80 | 0 | 0 | 0 | 5 | 0 | 0.5 | 0 | 20 |
| 60 | 0 | 0 | 0 | 40 | 0 | 0 | 0.1 | 10 | 10 |
| 14 | 56 | 15 | 15 | 0 | 1 | 0 | 0.1 | 0 | 20 |
| 60 | 0 | 0 | 0 | 40 | 0 | 0 | 0.5 | 20 | 20 |
| 18 | 52 | 30 | 0 | 0 | 0 | 0 | 0.1 | 20 | 10 |
| 17 | 52 | 0 | 30 | 0 | 0 | 0 | 0.3 | 0 | 20 |

| | | | | | | | | | |
|---|---|---|---|---|---|---|---|---|---|
| 80 | 0 | 0 | 0 | 20 | 0 | 0 | 0.1 | 10 | 10 |
| 80 | 0 | 0 | 0 | 20 | 0 | 0 | 0.5 | 20 | 20 |
| 17 | 53 | 30 | 0 | 0 | 0 | 0 | 0.1 | 10 | 20 |

**Table S2.** Statistics of the physical properties used in this study.

| Name | Young's Modulus | Tensile Strength | Elongation at Break | log Surface Resistivity | Density | Tg | Storage Modulus | Loss Tangent |
|---|---|---|---|---|---|---|---|---|
| Unit | MPa | MPa | % | | g/cm$^3$ | °C | Pa | |
| Number | 80 | 80 | 80 | 80 | 80 | 80 | 80 | 80 |
| Mean | 334.2 | 6.2 | 57.5 | 5.45 | 1.21 | 28.2 | 5.22E+08 | 0.63 |
| Standard Deviation | 599.3 | 6.2 | 48.1 | 3.43 | 0.09 | 25.7 | 1.08E+09 | 0.34 |
| Min | 1.6 | 0.9 | 1.4 | 0.11 | 1.01 | -44.1 | 1.40E+06 | 0.04 |
| Q1 | 9.3 | 2.1 | 21.5 | 2.72 | 1.15 | 18.0 | 1.54E+07 | 0.35 |
| Q2 | 85.0 | 4.2 | 41.7 | 4.52 | 1.22 | 33.2 | 1.16E+08 | 0.76 |
| Q3 | 324.1 | 7.3 | 81.4 | 7.37 | 1.28 | 45.5 | 5.08E+08 | 0.85 |
| Max | 2969.0 | 28.6 | 207.4 | 13.76 | 1.46 | 65.7 | 7.08E+09 | 1.25 |

**Table S3.** Effects of the input modality on the predictive performance of the MDL model.

| Name | Input Modalities | | | | Accuracies (Cross-validation:16 sets) | | |
|---|---|---|---|---|---|---|---|
| | IR | Raman | OM Image | Composition | $R^2$(Test) | RMSE(Test) | δRMSE (Test) |
| Benchmark | ● | ● | ● | ● | 0.70 | 0.538 | - |
| Remove IR Inputs | | ● | ● | ● | 0.68 | 0.549 | 0.012 |
| Remove Raman Inputs | ● | | ● | ● | 0.64 | 0.564 | 0.027 |
| Remove OM Image Inputs | ● | ● | | ● | 0.44 | 0.713 | 0.176 |
| Remove Composition Inputs | ● | ● | ● | | 0.38 | 0.733 | 0.195 |

δRMSE: difference of RMSE between the focused model and benchmark model.
